# ERBB3 influences the ferroptosis pathway via modulation of lipid peroxidation and GSH synthesis in gastric cancer

**DOI:** 10.1038/s41420-025-02707-2

**Published:** 2025-08-22

**Authors:** Robert Jenke, Theresa Heinrich, Florian Lordick, Achim Aigner

**Affiliations:** 1 Department of Medicine, Division of Oncology, University of Leipzig Medical Center, Comprehensive Cancer Center Central Germany (CCCG), Leipzig, Germany; 2 Leipzig University, Medical Faculty, Rudolf-Boehm-Institute for Pharmacology and Toxicology, Clinical Pharmacology, Leipzig, Germany

**Keywords:** Gastric cancer, Gastric cancer

## Abstract

Gastric cancer remains one of the most lethal malignancies worldwide, with high relapse rates and limited survival for patients with advanced disease. Despite advances in targeted therapies and immune checkpoint inhibition, intrinsic tumor heterogeneity poses challenges for effective treatment. The HER3 receptor (ERBB3) has emerged as an important player in cancer progression, contributing to aggressive tumor behavior and poor prognosis. Recent evidence indicates that activating ferroptosis—an iron-dependent, non-apoptotic form of cell death—offers a promising strategy to inhibit cancer growth. In gastric cancer, ferroptosis plays a crucial role, and promoting this process may open new avenues for therapeutic intervention. Ferroptosis is characterized by iron-mediated lipid peroxidation of cell membranes and is critically regulated by the cystine/glutamate antiporter system (SLC7A11) and glutathione peroxidase 4 (GPX4). Our study aimed to investigate the relationship between ERBB3 and ferroptosis in gastric cancer. We found that high ERBB3 expression correlated with resistance to ferroptosis-inducing agents, including GPX4 and SLC7A11 inhibitors, across multiple cell lines. Vice versa, ERBB3 inhibition with TX1-85-1 induced lipid peroxidation in gastric cancer cells, with effects most pronounced in cell lines expressing higher SLC7A11 levels. Knockdown of ERBB3 reproduced these effects, suggesting SLC7A11 as a predictive marker. Importantly, combined inhibition of ERBB3 and GPX4 significantly enhanced lipid peroxidation and cytotoxicity, while ERBB3 activation by co-treatment with the ERBB3 ligand heregulin reduced lipid peroxidation in cells with lower baseline SLC7A11 expression. Analysis of glutathione levels and SLC7A11 expression further supported the role of ERBB3 in modulating ferroptosis sensitivity. These findings identify ERBB3 as a critical regulator of ferroptosis and a promising target for enhancing ferroptosis-mediated cell death. Its inhibition in combination with ferroptosis inducers may thus represent a particularly promising and efficacious therapeutic strategy in gastric cancer.

## Introduction

Gastric cancer remains one of the most lethal malignancies worldwide, ranking as the fifth most common cancer with nearly one million new cases and 659,853 deaths in 2022 [1]. In the Western world, localized gastric cancer is typically managed with perioperative chemotherapy, which has been shown to improve overall survival outcomes. In contrast, East Asian countries favor curative surgery followed by adjuvant therapy [2]. Despite these treatment strategies, the risk of relapse remains high, and for patients with unresectable, locally advanced, or metastatic disease, the survival perspectives are very limited. Chemotherapy, consisting of agents such as fluoropyrimidines, platinum-based drugs, taxanes, and irinotecan, continues to serve as the cornerstone of treatment [3]. For over a decade, the addition of trastuzumab for HER2-positive gastric cancer was the primary option for targeted therapy [4]. However, recent advancements have introduced new treatment modalities. The approval of immune checkpoint inhibitors, such as nivolumab [5] and pembrolizumab [6] for PD-L1-positive cancers in combination with chemotherapy was based on the results of pivotal studies like CHECKMATE-649 and KEYNOTE-859. Additionally, combining trastuzumab, pembrolizumab and chemotherapy has been shown to significantly enhance progression-free survival and overall survival [6, 7]. The antibody-drug conjugate trastuzumab deruxtecan has demonstrated efficacy even in patients previously treated with trastuzumab [8], and most recently, the addition of zolbetuximab, a Claudin 18.2-targeting antibody, was approved for first-line treatment in combination with chemotherapy following the results of the GLOW [9] and SPOTLIGHT [10] trials. Nevertheless, the intrinsic inter- and intratumoral heterogeneity of gastric cancer continues to contribute to its poor prognosis, highlighting the urgent need for novel therapeutic strategies.

The HER receptor family, comprising ERBB1 (EGFR), ERBB2, ERBB3, and ERBB4, plays a pivotal role in tumorigenesis and cancer progression. These receptors can form homo- or heterodimers upon activation by a diverse array of ligands. Notably, ERBB3, despite lacking a functional kinase domain, transduces the strongest survival signals and is activated by the ligands heregulin-1 and -2 [11, 12]. ERBB3 expression has been associated with aggressive tumor features, including lymph node metastasis, and correlates with significantly worse overall survival outcomes [13].

In addition to the well-established mechanisms of programmed cell death, a form of iron-dependent, non-apoptotic cell death known as ferroptosis was first described by Dixon and colleagues in 2012 [14]. Recent evidence indicates that activating ferroptosis exerts anti-proliferative effects in gastric cancer. Therefore, it is of interest as a new targetable pathway for therapeutic intervention [15–17]. High expression of the anti-ferroptotic enzyme GPX4 has been linked to poor survival outcomes [18], while the SLC7A11 transporter may be crucial in the development of platinum resistance in gastric cancer [19]. Furthermore, upregulation of GPX4 has been identified as a key mechanism driving the progression of gastric adenocarcinoma, particularly in metastatic sites [20]. While there is some evidence suggesting that HER receptor signaling may influence ferroptosis in other cancer types, such as breast cancer [21] or melanoma [22], no studies to date have explored the potential link between ERBB3 and ferroptosis in gastric cancer.

## Results

### ERBB3 expression is associated with ferroptosis resistance

**Fig. 1:**
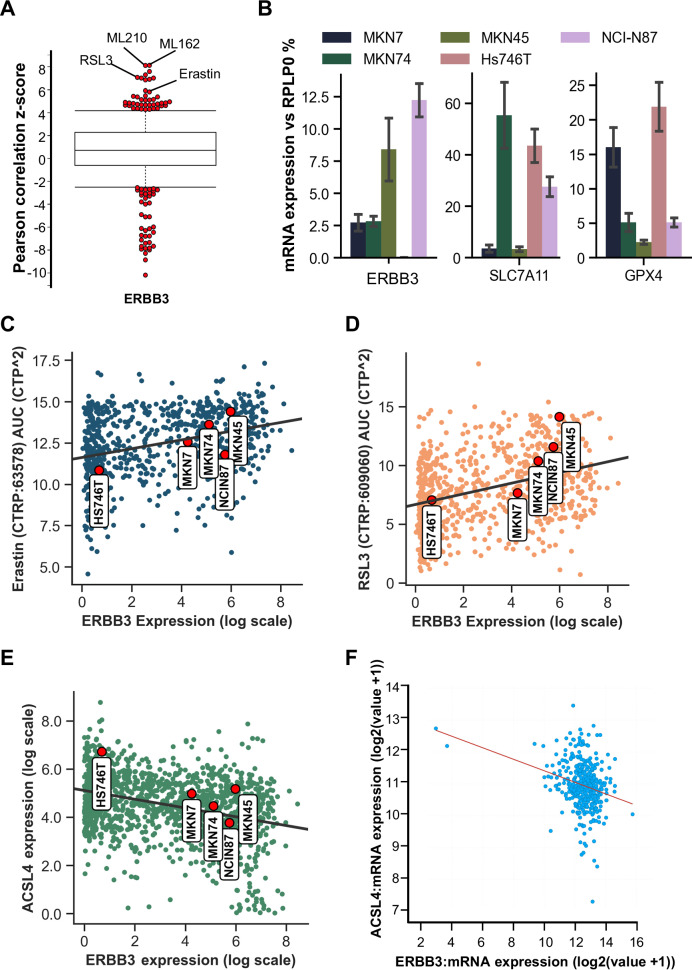
ERBB3 expression is associated with ferroptosis resistance. **A** Correlation analysis of ERBB3 expression with sensitivities to 481 compounds from the CTRP database v2. Z-scores: ML210 (8.11), ML162 (8.12), RSL3 (7.07), and Erastin (5.82). **B** mRNA expression analysis of ERBB3, SLC7A11, and GPX4 normalized to RPLP0 in five gastric cancer cell lines. **C** Correlation between Erastin area under the drug response curve (AUC) values from the CTRP database v2 and ERBB3 expression from the DepMap Expression 24Q2 dataset across 756 cell lines. Pearson coefficient: 0.319; *p* = 2.59E-19. **D** Correlation between RSL3 AUC values (CTRP database v2) and ERBB3 expression in 772 cell lines (DepMap Expression 24Q2 dataset). Pearson coefficient: 0.362; *p* = 2.86E-25. **E** Correlation between ACSL4 expression and ERBB3 expression in 1517 cell lines from the DepMap Expression 24Q2 dataset. Pearson coefficient: –0.354; *p* = 5.33E-46. **F** Co-expression analysis of ACSL4 and ERBB3 (RNA-Seq V2 RSEM) in 413 samples from the TCGA Firehose Legacy cohort. Pearson coefficient: –0.26; *p* = 1.46E-7.

To gain insights into the potential role of the ERBB3 receptor in the ferroptosis pathway, we conducted an initial screen using data from the Cancer Therapeutics Response Portal v2 (CTRPv2). We observed that ERBB3 expression was significantly associated with resistance against drugs targeting critical enzymes within the ferroptosis pathway, as indicated by high z-scores. Specifically, the z-scores for GPX4 inhibitors were particularly high: 8.11 for ML210, 8.12 for ML162, and 7.07 for RSL3. Additionally, ERBB3 expression correlated with reduced efficacy of Erastin (z-score 5.82), which acts as an inhibitor of the essential cystine transporter SLC7A11 (Fig. 1A).

For our study, we selected five gastric cancer cell lines with diverse genetic profiles—such as MET amplification in MKN45 cells and HER2 amplification in MKN7 cells—to represent the heterogeneity commonly observed in gastric cancer. ERBB3 expression levels were highest in MKN45 cells (8.4% relative to the housekeeping gene RPLP0), followed by MKN74 (7.14%), MKN7 (2.74%), and lowest in Hs746T cells (0.026%). SLC7A11 expression varied, with MKN7 (3.57%) and MKN45 (3.34%) displaying relatively low levels, whereas MKN74 (55.45%) and Hs746T (44.55%) exhibited significantly higher expression. GPX4 expression demonstrated variability across the cell lines as well (Fig. 1B).

Utilizing the DepMap Data Explorer, we visualized the CTRPv2 data to assess the relationship between Erastin area under the drug response curve (AUC) values and ERBB3 expression (AUC data available for 756 cell lines). Drug sensitivities of cell lines were derived as “area under the drug response curve” (AUC) as described previously (see e.g., Kurilov et al. [23]), which was found to be more accurate than the traditional metric of IC50 [24]. The correlation analysis revealed a moderate yet highly significant positive association (Pearson coefficient 0.319, *p* = 2.59E-19). This correlation was evident for the selected cell lines, with MKN45, exhibiting the highest ERBB3 expression, showing the highest AUC values for Erastin, while Hs746T, which had lower ERBB3 expression, showed a contrasting trend (Fig. 1C). The analysis of RSL3 AUC data across 772 cell lines revealed an even stronger positive association (Pearson coefficient = 0.362, *p* = 2.86 × E-25^−^). Notably, high ERBB3-expressing MKN45 cells exhibited an over 7-log-fold increase in RSL3 AUC compared to the low ERBB3-expressing Hs746T cells (Fig. 1D)

Finally, we examined ACSL4 expression, a well-recognized biomarker linked to ferroptosis sensitivity [25]. The analysis across 1517 cell lines available in the CTRPv2 dataset revealed a negative correlation between ERBB3 expression and ACSL4 levels (Pearson coefficient –0.354, *p* = 5.33E-46) (Fig. 1E). To corroborate these findings, we performed co-expression analysis using the Firehouse Legacy TCGA dataset, which includes 413 patient samples. Consistent with the cell line data, higher ERBB3 expression was associated with lower ACSL4 expression (Pearson coefficient –0.26, *p* = 1.46E-7, Fig. 1F). This data puts emphasis on high ERBB3 expression serving as a resistance factor against ferroptosis.

Collectively, these observations strongly support the involvement of ERBB3 in the ferroptosis pathway, including its potential implications in gastric adenocarcinoma.

### ERBB3 inhibition triggers lipid peroxidation in gastric cancer cell lines upon small molecule inhibition or siRNA-mediated knockdown

**Fig. 2:**
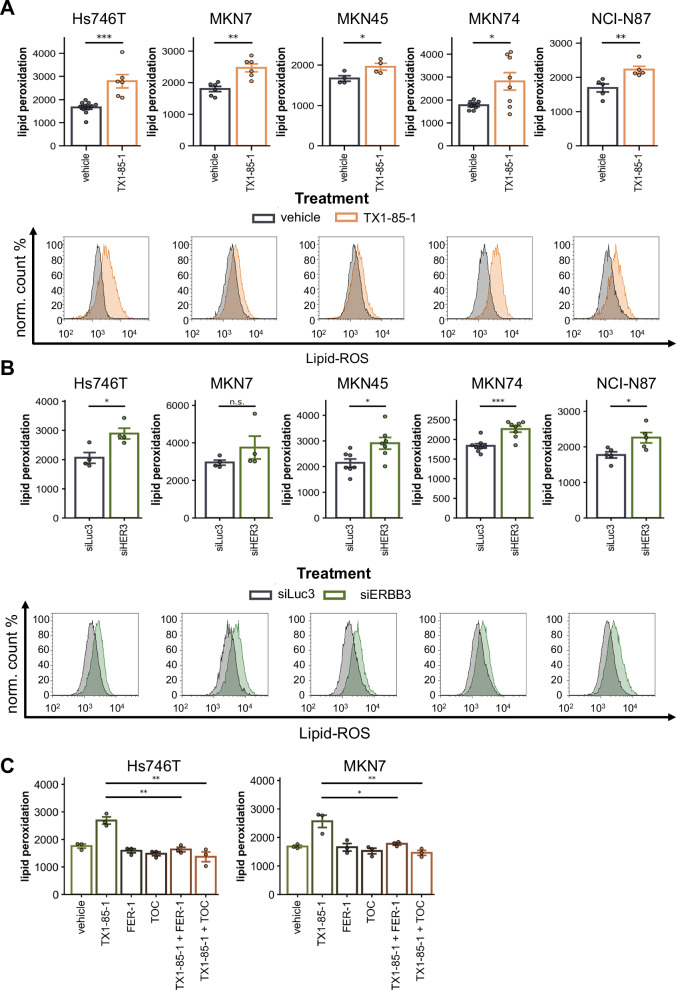
Lipid peroxidation induction upon ERBB3 inhibition or siRNA-mediated knockdown. **A** FACS analysis of lipid peroxidation via BODIPY™ 581/591 C11 staining. The upper panel summarizes data from ≥3 independent experiments, while the lower panel displays representative histograms. Cells were incubated for 48 h with TX1-85-1 (5 µM for Hs746T, MKN7, MKN45, and NCI-N87 cells; 3 µM for MKN74 cells). **B** Lipid peroxidation following siRNA-mediated knockdown of ERBB3 (20 nM siRNA). Cells were incubated for 72 h prior to analysis. **C** FACS analysis of lipid peroxidation via BODIPY™ 581/591 C11 staining from *n* = 3 independent experiments. Cells were incubated for 48 hours with 5 µM TX1-85-1, 1 µM Ferrostation-1, 100 µM α-Tocopherol, or the combination.

We treated five gastric cancer cell lines with the first reported selective ERBB3 inhibitor, TX1-85-1 [26]. Due to varying levels of toxicity, based on cell culture observations, the inhibitor was applied at concentrations of 5 µM for MKN7, MKN45, Hs746T, and NCI-N87 cells, and 3 µM for MKN74 cells. Following 48 h of treatment, we observed a significant increase in lipid peroxidation across all investigated cell lines (Fig. 2A). The most pronounced increase occurred in Hs746T and MKN74 cells, which exhibited approximately 1.6-fold higher levels of lipid peroxidation. Interestingly, these cell lines also demonstrated the highest baseline expression of SLC7A11. Wildtype SLC7A11 expression might correlate with sensitivity towards ERBB3 inhibition (see Supplementary Fig. 1).

In contrast, MKN7, MKN45, and NCI-N87 cells displayed more moderate increases in lipid peroxidation, with median fluorescence intensities elevated by approximately 1.2- to 1.4-fold. These findings align with their relatively low baseline levels of SLC7A11 expression (see Fig. 1B). In contrast to SLC7A11, the observed effects on lipid peroxidation were independent of baseline ERBB3 or GPX4 expression levels. Thus, wild-type SLC7A11 expression may serve as a predictive marker for lipid peroxidation increases in response to ERBB3 inhibition.

To validate these results, siRNA-mediated knockdown of ERBB3 recapitulated the lipid peroxidation effects induced by TX1-85-1 treatment (Fig. 2B). The knockdown efficacy across cell lines ranged from 65 to 92% in reducing ERBB3 mRNA levels. These findings collectively underscore the role of SLC7A11 expression in modulating lipid peroxidation responses to ERBB3 inhibition.

To validate our findings, we tested whether classic ferroptosis inhibitors (Ferrostatin-1) or lipid radical scavengers (α-Tocopherol) could reverse the observed phenotype. While baseline lipid peroxidation in Hs746T and MKN7 cells remained unchanged, both agents restored ERBB3 inhibition-induced lipid peroxidation to wild-type levels (Fig. 2C), supporting the role of ERBB3 in the ferroptotic pathway.

### Combined ERBB3 and ferroptosis inhibition enhances lipid peroxidation and cytotoxicity

**Fig. 3:**
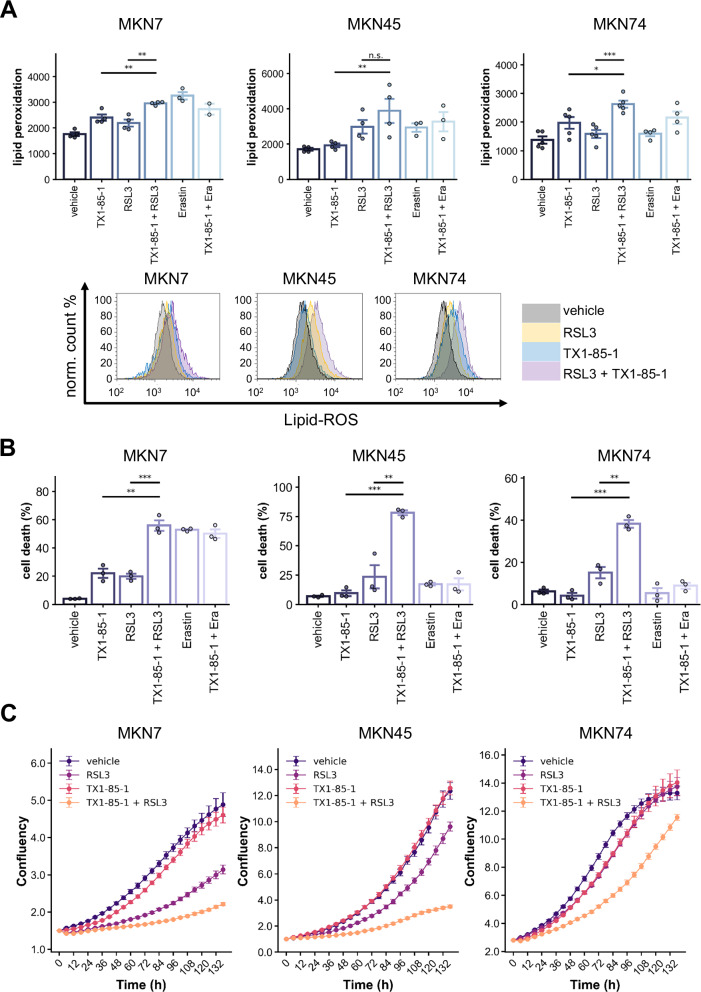
Combinatory effects of ERBB3 inhibition plus SLC7A11- or GPX4 inhibition on lipid peroxidation and cell viability. **A** FACS analysis of lipid peroxidation via BODIPY™ 581/591 C11 staining. The upper panel summarizes data from ≥3 independent experiments, and the lower panel presents representative histograms. Cells were treated for 48 h with RSL3 (4 µM), Erastin (10 µM), and TX1-85-1 (1 µM for MKN74, 5 µM for MKN7 and MKN45). **B** Cell death staining using propidium iodide from *n* = 3 independent experiments. Cells were treated for 48 h with RSL3 (4 µM), Erastin (10 µM), and TX1-85-1 (1 µM for MKN74, 5 µM for MKN7 and MKN45 cells). **C** IncuCyte^®^ proliferation assay depicting normalized confluency over 6 days. Cells were seeded in 96-well plates, and confluency was measured every 6 h. Treatment conditions: Erastin (1 µM), RSL3 (0.5 µM), and TX1-85-1 (2 µM for MKN45, 1 µM for MKN7, and 0.5 µM for MKN74).

We next explored the effects of combining ERBB3 inhibition with the ferroptosis inducers Erastin and RSL3 on lipid peroxidation levels and cell viability. Erastin demonstrated stronger effects in MKN7 cells (1.85-fold increase) and MKN45 cells (1.73-fold increase), which exhibit lower baseline SLC7A11 expression, compared to MKN74 cells (1.16-fold increase) (Fig. 3A).

Beyond TX1-85-1 single effects, the combination with Erastin showed only a non-significant lipid peroxidation increase in MKN74 cells. This applied as well for MKN45 cells with a more pronounced Erastin single effect. In MKN7 cells, the combination of Erastin with TX1-85-1 even reduced Erastin-induced lipid peroxidation.

In contrast, combining GPX4 inhibition via RSL3 with TX1-85-1 elicited a marked increase in lipid peroxidation across all three cell lines. This combination amplified the median fluorescence intensity (MFI) observed with RSL3 alone versus RSL3 with ERBB3 inhibition from 1.25-fold to 1.68-fold in MKN7, from 1.75-fold to 2.28-fold in MKN45, and from 1.16-fold to 1.91-fold in MKN74, compared to vehicle controls. The effect was statistically significant in MKN7 and MKN74 cells and exhibited a strong, though not statistically significant, trend in MKN45 cells (Fig. 3A).

We also investigated whether increased lipid peroxidation directly leads to cell death and found a strong correlation. In MKN7 cells, Erastin induced significant cell death, which was not further enhanced by TX1-85-1. RSL3 or TX1-85-1 alone caused ~20% cell death, but their combination tripled this effect. In MKN45 cells, RSL3 had a limited impact alone, yet combined GPX4 and ERBB3 inhibition led to ~75% cell death. Similarly, in MKN74 cells, the combination increased cell death up to ~38%. Erastin, alone or in combination with TX1-85-1, showed little effect in MKN45 and MKN74 cells (Fig. 3B).

Subsequently, we assessed the impact of the RSL3 and TX1-85-1 combination on cell viability using the IncuCyte^®^ system. While TX1-85-1 alone at 0.5–2 µM had minimal impact on cell viability—despite variability in sensitivity among cell lines—RSL3 treatment produced moderate effects in MKN7 and MKN45 cells. Strikingly, the combination of RSL3 and TX1-85-1 mirrored the lipid peroxidation findings and exhibited robust cytotoxic synergy (Fig. 3C). No additional effect was observed upon the combination of Erastin and TX-1-85-1 (data not shown).

In summary, this indicates that the capacity of SLC7A11 and ERBB3 inhibition to induce lipid peroxidation depends on wild-type SLC7A11 levels (without evidence of a synergistic interaction). Moreover, GPX4 inhibition with RSL3 combined with ERBB3 inhibition consistently augments lipid peroxidation, independent of expression levels, resulting in considerably higher cell death and decreased cell viability.

### ERBB3 stimulation with heregulin mitigates lipid peroxidation depending on the cell line

**Fig. 4:**
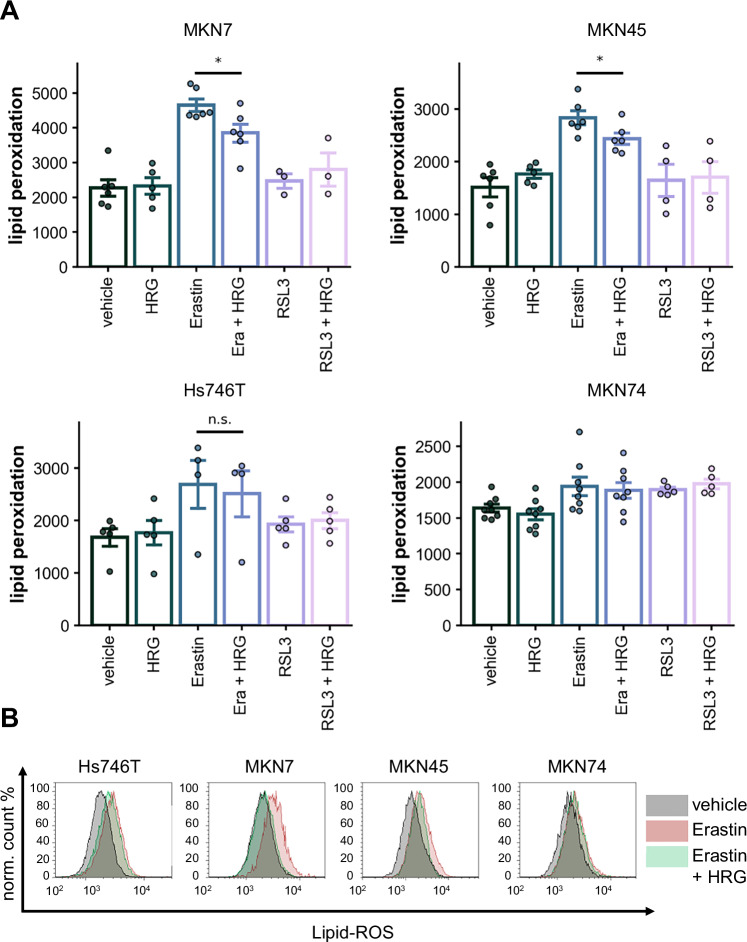
Rescue effects of ERBB3 stimulation with heregulin, diminishing lipid peroxidation. FACS analysis of lipid peroxidation via BODIPY™ 581/591 C11 staining. **A** Quantitation of data from ≥3 independent experiments, **B** representative histograms. Cells were treated for 48 h with RSL3 (4 µM for MKN7, MKN45, MKN74; 1 µM for Hs746T), Erastin (10 µM for all cell lines), and HRG (30 ng/ml).

To further validate our findings, we explored the reverse scenario by stimulating the cell lines with the specific ERBB3 ligand heregulin (HRG) during ferroptosis induction. Heregulin alone had no impact on baseline lipid peroxidation levels. However, co-treatment of MKN7 and MKN45 cells with both Erastin and heregulin significantly reduced Erastin-induced lipid peroxidation (Fig. 4A). This effect was not observed in Hs746T and MKN74 cells (Fig. 4B).

These results align with the SLC7A11 expression profiles (see Fig. 1B). MKN7 and MKN45 cells, which have lower baseline SLC7A11 expression, are more sensitive to Erastin-induced lipid peroxidation but are also more readily “rescued” by ERBB3 activation through heregulin. Conversely, cells with higher SLC7A11 expression, such as MKN74 and Hs746T, still show increased lipid peroxidation upon SLC7A11 inhibition. However, this higher baseline expression could make them less susceptible to the compensatory effects of ERBB3 activation by heregulin.

Interestingly, GPX4 inhibition with RSL3, which generally induces only modest increases in lipid peroxidation, remained unaffected by heregulin stimulation. This observation suggests that ERBB3 activation specifically modulates lipid peroxidation in the context of SLC7A11 inhibition but not through GPX4 activity.

### ERBB3 activation modulates SLC7A11 expression and glutathione levels

**Fig. 5:**
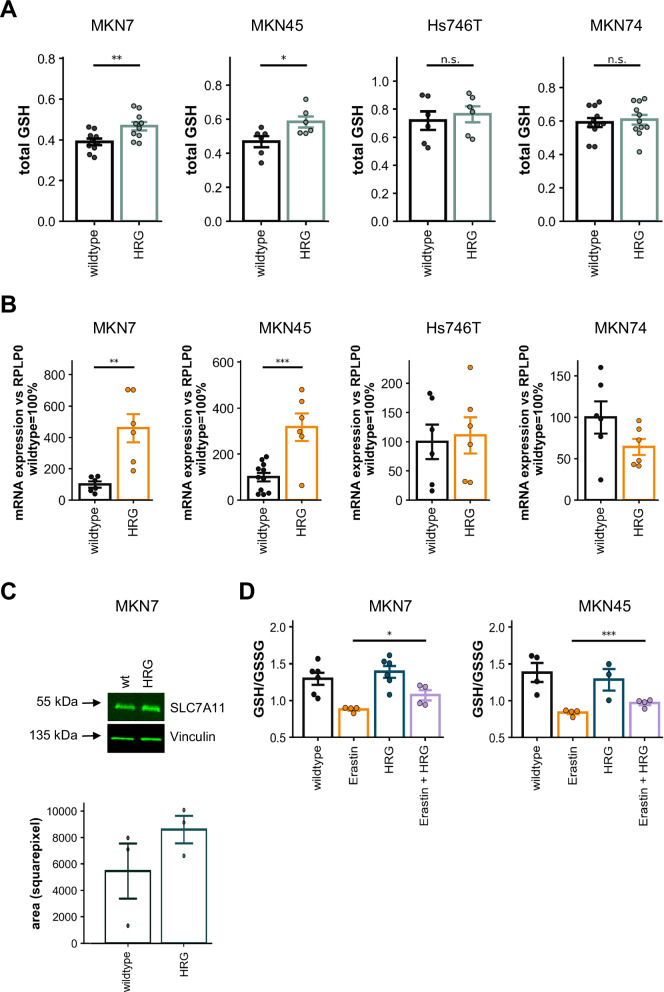
Alterations of SLC7A11 expression and glutathione levels upon ERBB3 stimulation. **A** Total glutathione (GSH) levels detected colorimetrically in cells treated with HRG (30 ng/ml) for 48 h. **B** mRNA expression levels of SLC7A11 normalized to the housekeeping gene RPLP0 (set to 100%) following treatment with HRG (30 ng/ml) for 48 h. **C** Western blot of SLC7A11 upon HRG stimulation for 48 h in MKN7 cells. Representative image of *n* = 3 experiments. **D** GSH/GSSG ratios in MKN7 and MKN45 cells treated with Erastin (10 µM) and HRG (30 ng/ml) for 48 h.

To further corroborate our above findings, we examined the total glutathione (GSH) levels across the cell lines. Consistent with the observed HRG-mediated rescue of lipid peroxidation in MKN7 and MKN45 cells, we detected an increase in total GSH levels following HRG stimulation (see Fig. 5A). Total GSH rose by ∼20% in MKN7 and ∼25% in MKN45 cells. In contrast, no significant change in total GSH levels was observed in Hs746T and MKN74 cells, which is in line with the lack of rescue effect on lipid peroxidation in these lines (see Fig. 4).

Further investigation into the mRNA expression of SLC7A11, the primary cystine transport channel essential for GSH synthesis, provided additional insights. HRG stimulation led to a substantial upregulation in SLC7A11 mRNA expression in MKN7 and MKN45 cells. The SLC7A11 mRNA levels upon HRG stimulation vs wildtype levels increased up to ∼460% in MKN7 and ∼320% in MKN45 cells. In contrast, HRG stimulation resulted in stable SLC7A11 expression levels in Hs746T cells, while a decrease was observed in MKN74 cells (Fig. 5B). The mRNA data were consistent with protein data in MKN7 cells, which showed again increased SLC7A11 expression upon HRG stimulation (Fig. 5C). This effect was less prominent in MKN45 cells and, in accordance with the mRNA levels, not seen in MKN74 cells (data not shown). Consequently, this translated to a partial rescue of the Erastin-induced reduction in the GSH/GSSG ratio in MKN7 and MKN45 cells upon HRG treatment, albeit wild-type levels were not fully restored (Fig. 5D). These results create a link between SLC7A11 and ERBB3 regarding ferroptosis sensitivity. Baseline expression levels might be of crucial importance.

## Discussion

Ferroptosis has emerged as a critical form of non-apoptotic cell death [14], with increasing relevance in cancer biology, alongside its established roles in neurological disorders and autoimmune diseases. While advancements in understanding the pathway—such as modifications in intracellular iron homeostasis and cellular lipid composition [27]—have broadened the field, the central mechanisms remain focused on cystine import via SLC7A11 and lipid radical detoxification through GPX4 [28].

Evidence highlights that evasion of ferroptosis can promote metastatic progression [20, 29] and confer resistance to chemotherapies like oxaliplatin and 5-FU, which are mainstays for advanced cancers without targetable options. Although numerous ferroptosis inducers have been identified [30], they face limitations. For instance, the first SLC7A11 inhibitor, Erastin [31], has poor metabolic stability and low water solubility, complicating its in vivo use. Similarly, sorafenib lacks both selectivity and potency [32].

ERBB receptors exhibit context- and subtype-specific roles in the regulation of ferroptosis across different cancer types. In melanoma and fibrosarcoma, overexpression of EGFR and ERBB2 sensitizes tumors to ferroptosis-inducing treatments through activation of the RAS/RAF/c-Myc/ACSL4 signaling axis [33]. In contrast, ErbB2 acts as a negative regulator in cervical cancer, where its upregulation suppresses sorafenib-induced ferroptosis via the Cdc25A/PKM2 pathway [34]. In breast cancer, the pan-ERBB inhibitor Neratinib (excluding ErbB3) enhances ferroptotic cell death [21, 35]. In melanoma, ERBB3 protects cells from ferroptosis by preserving antioxidant defenses, and its inhibition re-sensitizes resistant cells to RSL3 or Erastin treatment [22]. These findings collectively highlight that ERBB-mediated modulation of ferroptosis is highly dependent on both the specific receptor subtype and the tumor entity.

Regarding ERBB3, correlation analyses including data from CTRP associate its expression with resistance to ferroptosis-inducing drugs like Erastin and RSL3, based on AUC curves across various cancers, including gastro-esophageal cell lines. Although there are no FDA-approved ERBB3-specific therapies, promising clinical trials have demonstrated the safety and efficacy of ERBB3-targeting antibodies in solid tumors [36, 37].

This study is the first in gastric cancer to report that small-molecule inhibitors or siRNA-mediated knockdown of ERBB3 can induce lipid peroxidation. The effects are more pronounced in cells with high SLC7A11 expression. Interestingly, prior studies suggest that other members of the ERBB family, such as EGFR and ERBB2, may contribute to ferroptosis resistance [34, 38].

Our findings indicate that ERBB3 activation enhances glutathione levels and upregulates SLC7A11 expression. Notably, stimulation by heregulin induces nuclear translocation of NRF2, the master regulator of antioxidant responses, which directly drives SLC7A11 expression [39, 40]. While combining ERBB3 inhibition with Erastin may be redundant due to SLC7A11 inhibition by Erastin, targeting ERBB3 could additionally inhibit alternative cystine import pathways independent of SLC7A11.

Heregulin reduced oxidative stress in neurons exposed to H₂O₂, likely through upregulation of SLC1A1 [41, 42]. SLC1A1 independently transports cysteine into cells [43] and serves as a co-transporter with SLC7A11, importing glutamate in exchange for cystine [44]. This interplay between ERBB3 and SLC1A1 appears to involve HIF1α, which can be upregulated by heregulin and has been implicated in ferroptosis resistance in gastric cancer [45]. ERBB3 inhibition may therefore disrupt multiple cystine transport mechanisms, significantly increasing lipid peroxidation, especially when GPX4 activity is concurrently blocked. The increased lipid peroxidation observed with ERBB3 and GPX4 co-inhibition correlates with decreased cell viability. Previous studies have demonstrated cytotoxic effects of GPX4 inhibition only when combined with EGFR inhibition [46, 47]. Moreover, ferroptosis has been associated with gastric cancer progression, particularly at metastatic sites, highlighting GPX4 as a key mediator of resistance [20].

This study establishes a basis for combining ERBB3 inhibition with ferroptosis inducers like GPX4 inhibitors to achieve increased anti-tumor effects. By targeting multiple pathways simultaneously, this approach not only induces ferroptosis but may also prevent therapeutic resistance, providing new therapeutic options for treating advanced gastric cancer.

## Materials and methods

### Cell culture

Human gastric cancer cell lines MKN7, MKN74, MKN45, NCI-N87, and Hs746T were obtained from the American Type Culture Collection (ATCC, Manassas, VA, USA). Cell line authentication was performed regularly by genotyping (Genolytic, Leipzig, Germany). All MKN cell lines were cultivated in RPMI 1640 medium (Thermo-Fisher, Waltham, MA, USA) supplemented with 10% (*v*/*v*) heat-inactivated fetal bovine serum (FBS). The cell line Hs746T was cultivated in Dulbecco’s Modified Eagle’s Medium (4 mM L-glutamine, 4500 mg/L glucose, 1 mM sodium pyruvate, and 1500 mg/L sodium bicarbonate) supplemented with 10% (*v*/*v*) FBS. All media were used without antibiotics, and cells were kept at 37 °C in a humidified atmosphere containing 5% CO_2_ and passaged every 2–3 days. Erastin, RSL3, and TX1-85-1, Ferrostatin-1, and α-Tocopherol phosphate were purchased from Medchemexpress.

### Correlation analysis

The correlation between ERBB3 and ferroptosis inducers was analyzed using data from the Cancer Therapeutics Response Portal v2 [48–50]. Additional datasets were obtained via the DepMap Data Explorer, leveraging the latest DepMap Release (24Q2), including CRISPR screens, PRISM drug screens, copy number alterations, mutations, gene expression profiles, and gene fusions. The dataset is cited as: DepMap, Broad (2024). DepMap 24Q2 Public. Figshare+. Dataset. 10.25452/figshare.plus.25880521.v1. Data processing and visualization were conducted using Python 3.12, with libraries including Pandas (v2.2.2), Seaborn (v0.13.2), and Matplotlib (v3.8.4). Clinical data were sourced from the Stomach Adenocarcinoma (TCGA, Firehose Legacy) cohort. The findings presented are partially or entirely derived from data generated by The Cancer Genome Atlas (TCGA) Research Network: https://www.cancer.gov/tcga. Access to the TCGA data was facilitated through cBioPortal [51–53].

### Cell transfection and treatment

siRNAs were purchased from Eurofins MWG Operon (Ebersberg, Germany). In all knockdown experiments, irrelevant siRNA targeting luciferase (pGL3) was used as a negative control. Prior to transfection, cells were seeded in 12-well cell culture plates and maintained overnight under standard conditions. 20 nM siRNA was transfected using INTERFERin (Polyplus, Illkirch, France) at 1 μL INTERFERin™/pmol siRNA according to the manufacturer’s protocol. For sequences see Supplementary Table S1.

### Lipid peroxidation staining

Lipid peroxidation levels were analysed using the Image-iT Lipid Peroxidation Kit with BODIPY 581/591 C11 as a sensor according to the manufacturer’s instructions (Thermo-Fisher). In brief, cells were washed with PBS, trypsinized, and pelleted for 5 min at 100 g. Cells were then resuspended in growth medium containing BODIPY 581/591 C11 and stained for 30 min in the dark at 37 °C. Upon staining, cells were washed twice and then resuspended in PBS for measurement. FACS analysis was carried out using an Attune Acoustic Focusing Cytometer with Attune Cytometric Software.

### Measurement of cell death

Cells were treated at the indicated inhibitor concentrations and time points and then washed with PBS, trypsinized, and pelleted for 5 min at 100 g. The cells were stained with 100 ng/ml propidium iodide (Carl Roth, Karlsruhe, Germany) for 5 min at room temperature and then washed twice and resuspended in PBS. FACS analysis was carried out using an Attune Acoustic Focusing Cytometer with Attune Cytometric Software.

### Proliferation assay

For IncuCyte^®^ proliferation assays, cells were seeded in 96-well plate (100 cells/well) and cultured in absence or presence of drugs at indicated concentrations. Cell confluence was measured every 6 h (4 pictures/well) and quantified by the IncuCyte^®^ imaging system (Essen Bioscience).

### RNA isolation and RT-qPCR

Total RNA from cells was isolated using the guanidinium thiocyanate–phenol–chloroform extraction procedure (my-Budget RNAmagic, Bio-Budget Technologies, Krefeld, Germany). The first-strand synthesis was carried out using RevertAid™ H Minus First Strand cDNA Synthesis Kit (Thermo Fisher Scientific). Products were amplified using specific, intron-spanning primer pairs with RPLP0 serving as loading control primers (for primer sequences, see below). Real-time PCR was performed using the PerfeCTa SYBR Green FastMix® from QuantaBio (Beverly, MA, USA). One µl of each primer pair (5 µM) and 4 µl from the 1:10 prediluted first-strand synthesis were added to the reaction mixture (10 µl total), and the PCR was carried out in a StepOnePlus™ Real-Time PCR System (Thermo-Fisher) using the following conditions: 15 min of initial strand denaturation at 95 °C, followed by 55 cycles of 10 s at 95 °C, 10 s at 55 °C, and 10 s at 72 °C each. Fluorescence intensities were recorded in each cycle after the extension step at 72 °C. Crossing points were determined by the StepOne™ Software version 2.3. For Primer sequences see Supplementary Table S1.

### Glutathione measurement

For the determination of glutathione levels, we used the Glutathione Colorimetric Detection Kit (Invitrogen™, Thermo Fisher Scientific). Cells were seeded into 12-well plates and incubated for 48 h at the indicated drug concentrations. Cells were lysed with 5% 5-sulfo-salicylic acid and multiple freeze-thaw passages. For determining the oxidized glutathione content, samples were treated for 1 h with 2-vinylpyridine. Subsequently, samples were processed with Reaction Mixture and Detection Reagent according to the manufacturer's protocol. The absorbance at 405 nm was measured using a Multiskan™ FC Microplate Photometer (Thermo Fisher Scientific, Schwerte, Germany).

### Western blot

For Western blot analysis, cells were seeded at 80,000–100,000 cells/well (dependent on the cell line) in six-well plates. The next day, compounds were added and cells were treated for the indicated time periods. Prior to lysis, cells were washed with ice-cold phosphate-buffered saline, and then lysed in 30 µl of RIPA lysis buffer containing 1:200 Protease Inhibitor Cocktail Set III (EMD Millipore Corp, Billerica, MA, USA). After determination of protein concentrations using Thermo Scientific™ Pierce BCA Protein Assay Kit (Thermo-Fisher), lysates containing 25 µg total protein were mixed with loading buffer (125 mM Tris (pH 6.8), 20% glycerol, 4% SDS, 2% β-mercaptoethanol, 10 µg/mL bromophenol blue). After heat denaturation, the mixture was loaded onto 8% SDS-polyacrylamide gels and run at 80 V for 30 min and then at 120 V for 60 min, prior to electroblotting onto nitrocellulose membranes using Trans-Blot® Turbo™ Transfer System (Bio-Rad Laboratories, Germany). To control for equal loading, the blotting membrane was stained with Revert™ 700 Total Protein Stain according to the manufacturer’s instructions. After destaining, the blots were incubated for 1 h in 5% milk in TBST (150 mM NaCl, 100 mM Tris, pH 7.4, 0.05% Tween-20) to saturate non-specific binding sites, washed three times in TBST, horizontally cut and incubated with the specific primary antibody (anti-SLC7A11/xCT Polyclonal 1:1000 or anti-Vinculin Monoclonal 1:5000) at 4 °C overnight on a roller shaker. Anti-SLC7A11/xCT Polyclonal (Cat No. 26864–1-AP) was purchased from Proteintech (Planegg-Martinsried, Germany), and anti-Vinculin was from Sigma-Aldrich (Taufkirchen, Germany; Cat No. V9131). The next day, blots were washed three times with TBST, prior to incubation with IRDye® 800CW secondary antibody (LI-COR Corporate, Lincoln, NE, USA; 1:20,000 in 5% milk/TBST) for 1 h in the dark. After washing as above, near infrared signals on membranes were recorded using an Odyssey® XF Imaging System (LI-COR Corporate, Lincoln, NE, USA). Image acquisition was carried out by Empiria Studio® Software, and quantification was performed with ImageJ using vinculin as a loading control.

### Use of large language models

AI-assisted copy-editing was used for improvements to human-generated texts for readability and style, and to ensure that the texts are free of errors in grammar, spelling, punctuation, and tone. We used ChatGPT version 3.5.

### Bioinformatics analysis and statistics

All assays were performed independently at least three times unless indicated otherwise, and either one representative experiment or means ± s.e.m. of multiple experiments are shown. Statistical significance of differences in all assays was assessed by two-sided *t*-test in SigmaPlot 13, with *, <0.05; **, <0.01; and ***, <0.001.

## Supplementary information

Supplementary Table S1

Supplementary Fig. 1

Uncropped Western blots

## Data Availability

The datasets generated and/or analysed during the current study, if not included in this published article and its supplementary information files, are available from the corresponding author on reasonable request.
