# Supplementary material for: ERBB3 influences the ferroptosis pathway via modulation of lipid peroxidation and GSH synthesis in gastric cancer
Source: Cell Death Discov. 2025 Aug 22;11:398. doi: 10.1038/s41420-025-02707-2 (PMC12373893; doi:10.1038/s41420-025-02707-2)
Supplement: Supplementary file 1 — Supplementary Table S1 [file 41420_2025_2707_MOESM1_ESM.docx]

Suppl. Table S1

siRNA sequences

| **Target** | **Nucleotide sequence (5’ – 3’)** | |
| --- | --- | --- |
| ERBB3 | sense | CCUUGAGAUUGUGCUCACGdTdT |
|  | antisense | CGUGAGCACAAUCUCAAGGdTdT |
| pGL3 | sense | CUUACGCUGAGUACUUCGAdTdT |
|  | antisense | UCGAAGUACUCAGCGUAAGdTdT |

RT-qPCR Primer sequences

| **Target** | **Forward primer sequence** **(5' 🡪 3')** | **Reverse primer sequence (5' 🡪 3')** |
| --- | --- | --- |
| RPLP0 | TCTACAACCCTGAAGTGCTTGAT | CAATCTGCAGACAGACACTGG |
| GPX4 | ACAAGAACGGCTGCGTGGTGAA | GCCACACACTTGTGGAGCTAGA |
| SLC7A11 | TCCTGCTTTGGCTCCATGAACG | AGAGGAGTGTGCTTGCGGACAT |
| ERBB3 | CTGATCACCGGCCTCAAT | GGAAGACATTGAGCTTCTCTGG |
