# Supplementary material for: ERBB3 influences the ferroptosis pathway via modulation of lipid peroxidation and GSH synthesis in gastric cancer
Source: Cell Death Discov. 2025 Aug 22;11:398. doi: 10.1038/s41420-025-02707-2 (PMC12373893; doi:10.1038/s41420-025-02707-2)
Supplement: Supplementary file 2 — Supplementary Fig. 1 [file 41420_2025_2707_MOESM2_ESM.pptx]

## Slide 1
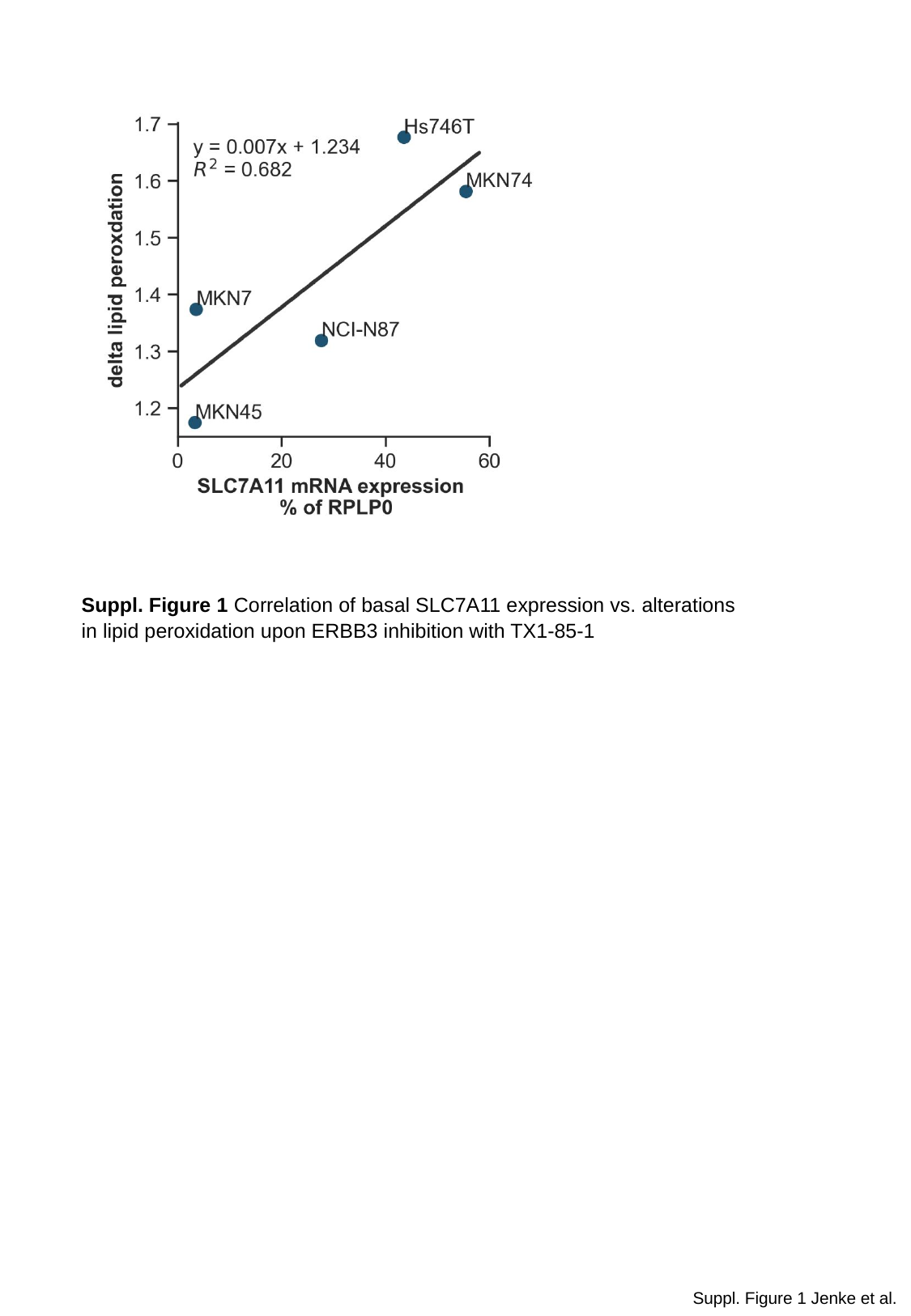

Suppl. Figure 1 Correlation of basal SLC7A11 expression vs. alterations in lipid peroxidation upon ERBB3 inhibition with TX1-85-1
Suppl. Figure 1 Jenke et al.
