# Supplementary figures and images for: ERBB3 influences the ferroptosis pathway via modulation of lipid peroxidation and GSH synthesis in gastric cancer
Source: Cell Death Discov. 2025 Aug 22;11:398. doi: 10.1038/s41420-025-02707-2 (PMC12373893; doi:10.1038/s41420-025-02707-2)

## Slide 1
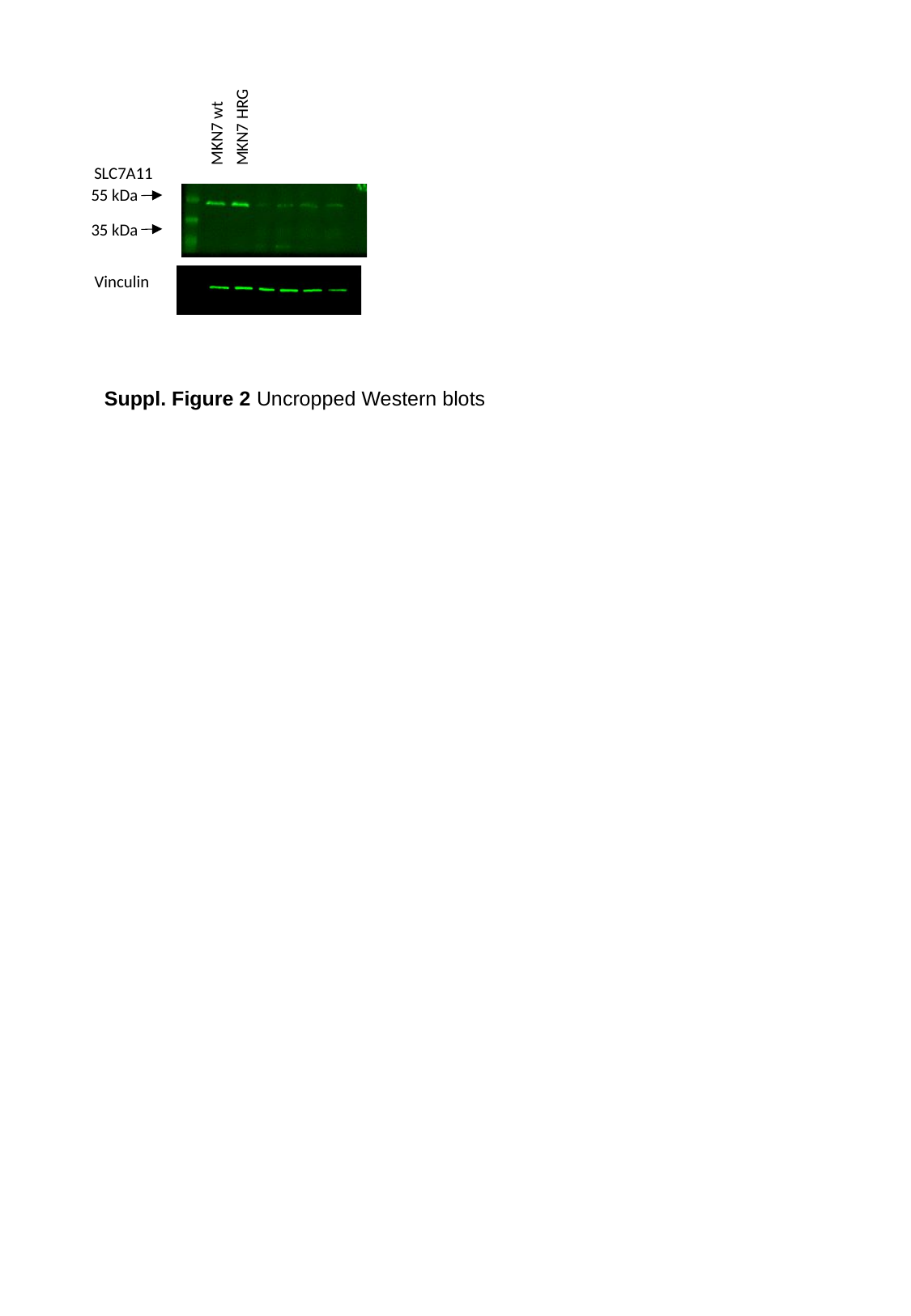

MKN7 HRG
MKN7 wt
SLC7A11
55 kDa
35 kDa
Vinculin
Suppl. Figure 2 Uncropped Western blots

Supplement: Supplementary file 3 — Uncropped Western blots [file 41420_2025_2707_MOESM3_ESM.pptx]
